# Rapid-Test Kit for Cardiac Troponin I: A Reliable Enzyme-Linked-Immuno-Substrate-Assay-Based Biosensor for Daily-Use Naked-Eye Detection and Pharmacokinetic Studies for Myocardial Infarction in Cardiovascular Disease

**DOI:** 10.1021/acsptsci.4c00218

**Published:** 2024-07-15

**Authors:** Yu-Fang Hsieh, Kuan-Jiuh Lin

**Affiliations:** † La Morongo Co. Laboratory, Berkeley, California 94720, United States; ‡ Department of Chemistry, National Chung Hsing University, Taichung City 402, Taiwan

**Keywords:** cardiovascular disease, myocardial infarction, cardiac troponin i, rapid-test kit, pharmacokinetic, portable test strip, home-diagnosis, combinational drug, enzyme-linked-immuno-substrate-assay (ELISA), artificial intelligence (AI)

## Abstract

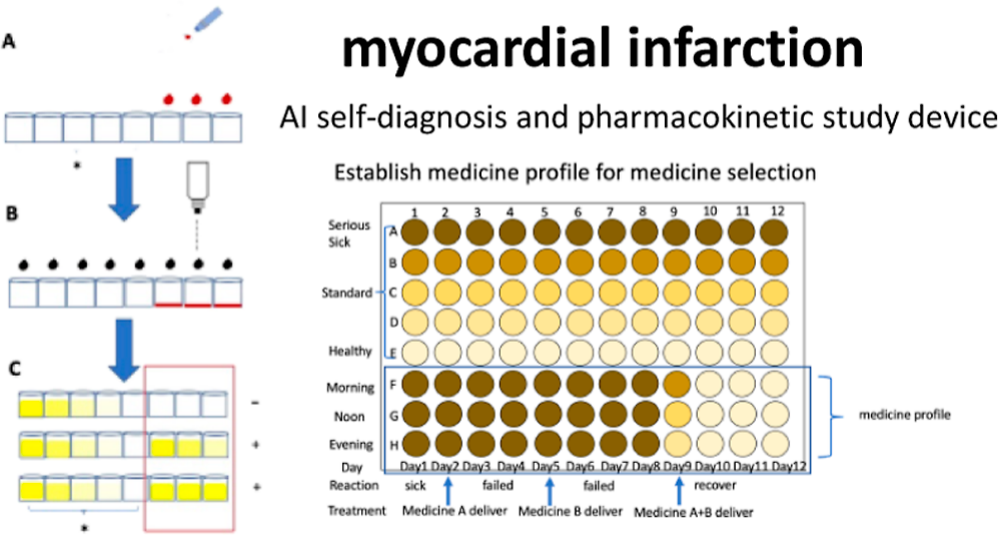

Myocardial infarction (MI) is a severe cardiovascular event that can lead to death. Cardiac troponin I (cTnI) is an MI biomarker in the circulation system; however, methods for detecting cTnI protein require substantial time, tedious operations, an expensive reader for translating signals, and a lot of reagents. This study aims to create a cTnI protein test kit with results easily distinguished by color differences, explicitly focusing on the resolution between different concentrations that eyes can discern. These results will aid in creating a commercial, portable, convenient, daily-use rapid-test kit. This study proposes a cTnI biosensor that the naked eye can perceive, performs diagnoses based on pattern color, does not require a reader machine, is easy to operate, and is portable. Our device shortens diagnosis time, has a 0.32–200 ng/mL quantitative analysis range in the human serum matrix, achieves a 0.32 ng/mL limit of detection, and exhibits many advantages compared to a traditional cTnI ELISA plate.

## 1.

In cardiovascular diseases (CVDs), plaque accumulates in the coronary artery and affects blood flow,^1−9^ resulting in a lack of oxygen or nutrition in the heart that can lead to death. The American Heart Association annually releases statistical reports on heart disease, stroke, and cardiovascular risk factors, and considerable research efforts are devoted to CVD.^10,11^ This focus includes artificial polymer bypass graft transplants,^7,12−21^ stent material innovations, antithrombosis medicine studies,^12,22−33^ tissue engineering for artificial vascular grafts,^13,19,60^ tissue repair medicine for ischemia,^8,34−39^ and creating biomarkers for detection.^8,33,36,40^

Myocardial infarction (MI) refers to myocardial cell death due to ischemia or the unbalanced supply within coronary arteries. There are six MI types.^36^ Excluding type 3, cardiac arrest, all other MI types can be evaluated based on biomarkers in blood circulation. “What is a “good” cardiac biomarker?” Cardiac biomarkers must comprise a protein with a high concentration in the myocardium only, be rapidly released into the circulation system, and be present for a sufficiently extended period of time while myocardial necrosis occurs.^36^ A biomarker should be easily detectable via a relatively inexpensive, straightforward, and designable assay. Several biomarkers used in CVD diagnosis include creatinine kinase, myoglobin, and cardiac troponin. Cardiac troponin is the most commonly used biomarker with the highest known sensitivity. It enters the bloodstream after a heart attack and remains days after all other biomarkers return to normal levels.^40−43^

In 2000, the European Society of Cardiology (ESC) and the American College of Cardiology employed cardiac troponin, a cardiac biomarker, for MI diagnosis. Contrasting many previous cardiac biomarkers utilized for point-of-care testing,^36^ cardiac troponins are regulatory proteins that control the calcium-mediated interaction of actin and myosin.^10,40−43^ Since MI diagnosis requires increasing or decreasing troponin concentrations, designing a detector that records dynamic troponin patterns is exceptionally useful and can assist in determining the diagnosis. In other words, detecting troponin patterns can help differentiate MI from other elevated troponin etiologists, preventing diagnostic misclassification.^36,44,45^ Although numerous researchers have designed simple and quantifiable sensors to detect cardiac troponins,^46^ most focus on accuracy. No existing sensor can distinguish cardiac troponins based on pattern color changes.

Furthermore, most sensors require readers, machines, and a meticulous reagent and structural arrangement.^8,36,47^ An electrochemical chip requires a fine nanostructure or quantum dot arrangement, extensive fabrication, high costs, and lengthy detection time. A fluorescent chip has a slightly shorter detection time than an electrochemical chip but requires a costly fluorescent detector. In comparison, a colorimetric chip employing horseradish peroxidase and 3,3′,5,5′-tetramethylbenzidine (HRP-TMB) as an enzyme–chromophore pair is more convenient, has a shorter analysis time, and is lower in cost. Immunosensors, enzyme-linked immunosorbent assays (ELISA), have an extended history of detecting biomolecules specifically, precisely, and with high selectivity.^33,46,48−58^ However, traditional HRP-TMB platforms require costly UV–vis detectors. Contrasting traditional HRP-TMB platforms and investigating the coating times and quantities for different layers, the proposed device developed herein uses HRP-TMB and does not require a detector. This study aims to create a device for detecting cardiac troponin I (cTnI) based on “naked-eye detection.” This device is easy to use, low-cost, and presents color patterns for diagnosis that can be used for pharmacokinetic studies and daily self-diagnoses at home.^59^

## 2 Materials and Methods

### 2.1 Fabrication of cTnI Naked-Eye Detection Well Strips

#### 2.1.1 Coating the cTnI Primary Antibody (1Ab) on the Bottom of Each Well for the First Layer

The 96-well plate (SIWARD LSPR02 or Corning 3596 clear bottom high-bind 96-well plate) is assembled. The cTnI primary antibody (1Ab) stock solution is prepared as follows: 2 μg/mL monoclonal mouse anti-cTnI (HyTest 4T21 cm^3^, 25 kDa) is dissolved in phosphate-buffered saline (PBS), and a multichannel pipet was used to add 100 μL/well. Next, this was incubated using the super adsorption machine (SIWARD Crystal) at 15 W for 20 min. The 96-well plate was rotated 180° after 10 min to prevent uneven coating on the well edges. Alternatively, this procedure can be performed via incubation at room temperature for over 1 h. The 1Ab coating goal is approximately 0.1 μg/well: 0.07065 cm^2^/well and 7.065 ng/cm^2^ area coverage. The maximum loading quantity is 1 μg/well; higher values will cause oversaturation and yield a considerably detached second layer since antibody-protein binding requires space. Lastly, the solution is removed and each well is washed 3–4 times with 1× PBS tween (PBST, Sigma-Aldrich 3563) using a Wellwash machine (Thermo Fisher).

#### 2.1.2 Blocking and Preventing Nonspecific Binding

A blocking buffer comprising 5% (w/v) bovine serum albumin (BSA)/PBST was prepared, and 100 μL of blocking buffer was added into each well with a multichannel pipet. The plate was covered with aluminum foil and incubated for 1 h at room temperature. The solution was removed, and each well was washed 3–4 times with 1× PBST.

#### 2.1.3 Coat Standard Protein cTnI as the Second Layer

A standard cTnI protein stock solution (1 μg/mL cTnI; recombinant protein human cTnI, HyTest/8RT17, 24 kDa) was prepared in a PBS buffer and serially diluted to 8, 1.6, 0.32, and 0.064 ng/mL. One well strip was taken. From the top to the bottom well (wells A–H), 8, 1.6, 0.32, 0.064, 0, 0, 0, and 0 ng/mL of cTnI protein solutions were added, respectively. This step can be expanded from 1 to 12 strips (or more) relative to requirements. The plate was covered with aluminum foil and incubated for 2 h at room temperature. The solution was removed, and each well was washed 3–4 times with 1× PBST.

#### 2.1.4 Airtight Packaging for Storage

The solution was removed from the well, and three well strips were placed into a vacuum bag sealed and packed under −76 cmHg for 10 s, and then the well strips should be stored in a fridge at 4 °C. The self-life of the wells is 7 months under proper airtight and fridge storage.

### 2.2 cTnI Naked-Eye Detection Well Strips for Test Patients, Healthy Humans, and Matrix Effect

#### 2.2.1 Preparation of 15 mL of Healthy Human Serum (Millipore S1–100 mL) and Patient Samples

Three spike samples (served as patient samples) were prepared as follows: cTnI standard protein was dissolved and serially diluted in three healthy human serums with concentrations: *T*1 = 0.08, *T*2 = 0.64, and *T*3 = 1.28 ng/mL.

#### 2.2.2 Opening the cTnI Naked-Eye Detection Well Strip Package and Taking One Strip for Testing

100 μL of healthy human serum was added per well on the well strip for the wells A–E. Then, *T*1, *T*2, and *T*3 were added for wells F–H, respectively (for testing real samples, pricking blood samples within 100 μL of PBS can be added here instead). All wells were incubated for 30 min, and the wells were washed 3–4 times with PBST (deionized or normal water can also be used). *Another “10× dilute human serum” means: the patient samples are diluted ten times by PBS (Reagent A) to reach the cTnI concentration at *T*1′ = 0.008 ng/mL, *T*2′ = 0.064 ng/mL, and *T*3′ = 0.128 ng/mL concentrations.

#### 2.2.3 Preparation of the cTnI Secondary Antibody Linked by the HRP Stock Solution (2Ab-HRP)

Monoclonal mouse anti-cTnI (HyTest 16A11) was added to 0.37 μg/mL (reagent B), and a multipipette was used to add 100 μL into each well. All wells were incubated for 30 min at room temperature; then, the wells were washed with 1× PBST 3–4 times (deionized or normal water can also be used).

#### 2.2.4 Addition of TMB (Reagent C)

100 μL of 1× TMB (3,3′,5,5″-tetramethylbenzidine; Sigma-Aldrich) was added into each well, incubated for 10 min, and quenched with 50 μL of 2 N H_2_SO_4_ (reagent D). The yellow color intensity was checked, and well F–H was compared with the standard color (well A–E) to track the patient’s serum’s cTnI concentration pattern. To compute and get the exact amount, the value shown by the color code was multiplied by the conversion factor of 15 ± 7.14 for human serum and 1.84 ± 0.76 for 10× dilute human serum. To diagnose MI, the color pattern in wells F–H was checked to see if color increased.

#### 2.2.5 Measurement of UV Absorbance

A UV detector was used to measure absorbance at 450 and 620 nm to calculate the amount of cTnI. A620 was subtracted from A450, and the result is plotted against cTnI concentration. The calibration curve was established, and each well’s value was recorded.

#### 2.2.6 Design for Public Use

Reagent A = PBS or deionized water; reagent B = 2Ab-HRP; reagent C = TMB; reagent D = 2N H_2_SO_4_; and reagent E = washing buffer = PBST. Reagent ABCDE and airtight well strips will be commercially prepared for public use. For public use, prick blood into well strips and then add reagents A, B, C, D, and E in series in the proper timing (see Figure 6).

## 3 Results and Discussion

MI is a severe cardiovascular event identified clinically, such as through the use of an electrocardiogram and cTnI biomarkers. However, sometimes severe cardiovascular events remain unknown and require novel recordings of cTnI biomarker patterns. The proposed portable cTnI diagnosis device can be used for self-diagnosis daily at home or in pharmacokinetic and pharmacodynamic clinical case studies. We designed and created a prototype implementing color patterns to visualize cTnI protein with the naked eye for a portable self-diagnosis device at home, pharmacokinetic study, and medicine targeting in a clinical setting. The sandwich-ELISA-based model was used to design a naked-eye cTnI protein device: bottom layer, primary cTnI antibody; middle layer, cTnI standard protein; top layer, secondary cTnI antibody. The selection of secondary antibody, most efficient binding duration, and amount of each layer are analyzed in the following sections.

### 3.1 Gold Secondary Antibody Tested in This Sandwiched Immunosensor Indicating Nonlinear Correspondence but Showing a Decrease in Standard Deviation (SD), Which is Evidence to Show that Studying Dilution Ratio is a Direction to Design the Device

**Figure 1:**
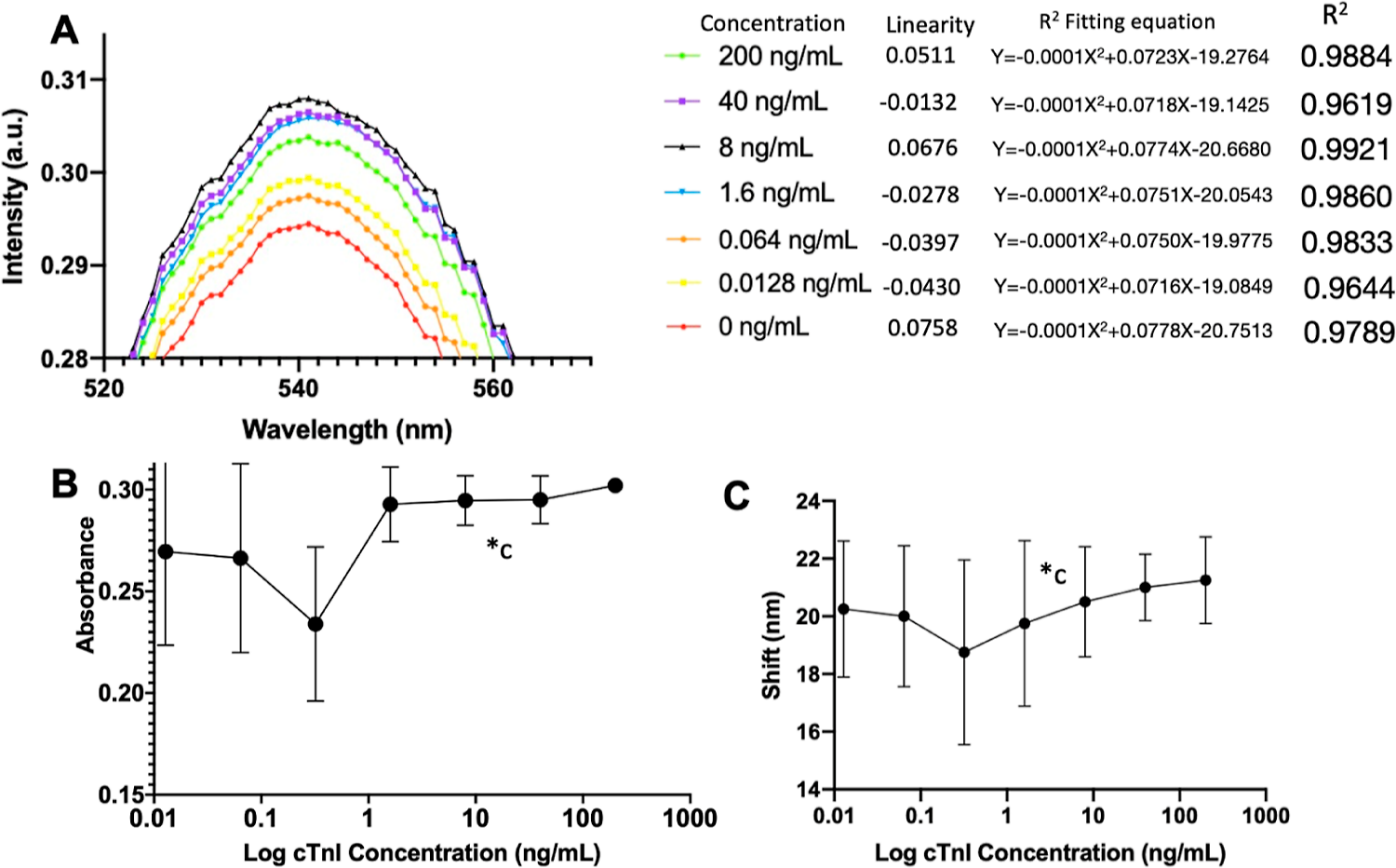
Gold secondary antibody was employed for cTnI detection, as shown in the plot: (A) entire spectrum absorption. The linearity for the different concentrations are all close to 0, indicating the wavelength and intensity do not have a linear correlation. While *R*^2^ is larger than 0.95, indicating all spectra are good fits in the quadratic curve; (B) absorption following blank-plate background correction, and (C) red shift vs log cTnI concentration. Indicate *c, the error bar decreased as cTnI concentration increased, indicating that studying an antibody’s dilution ratio can enhance resolution.

Gold nanoparticles and HRP can both serve as secondary antibodies and are valuable tools for the naked eye detection of cTnI due to their color-changing properties. Lin et al.^45^ developed the gold hot spot method, wherein Au nanoparticles (approximately 15 nm in diameter) were aggregated and clustered on top of an antibody, increasing the Au particles’ diameter. When well bottoms are coated by another Au particle layer, these Au layers induce a wavelength shift, known as the localized surface plasmon resonance (LSPR) effect. As shown in Figure 1A, standard cTnI protein levels increase at 540 nm absorbance. The quantity of nonlinear changes at this wavelength complicates data analysis and remains after background absorbance correction (Figure 1B). Therefore, we altered the analysis by considering 550 nm as the origin point and plotting the wavelength shift against the cTnI concentration logarithm.

**Figure 2:**
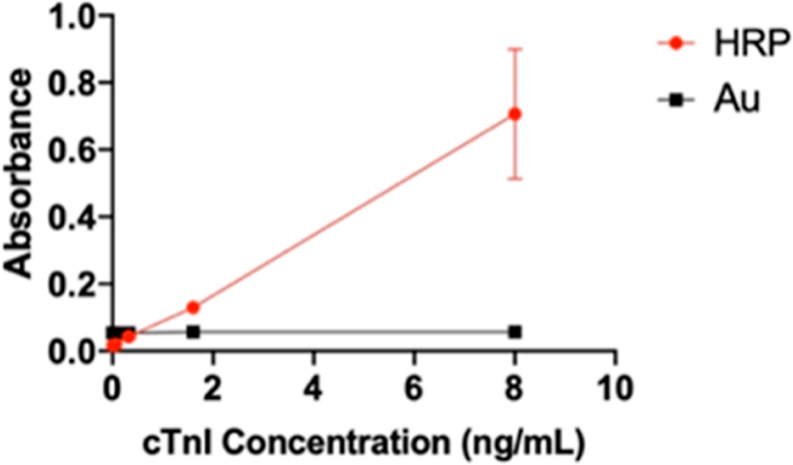
Gold hot spot 2Ab vs HRP-2Ab. HRP-2Ab has a better linear response and calibration line than the Au-2Ab method.

This analysis method successfully created a linear slope with a 0.5–1000 ng/mL cTnI dynamic range. The red shift peak trend confirmed LSPR signaling (Figure 1C), meaning the cTnI protein concentration increased as the wavelength changed. However, the wavelength-color difference was still not significant enough to be distinguished by the naked eye. Thus, the secondary antibody HRP-2Ab was selected instead. In addition, the decreasing error bar in Figure 1B (note c) proves that studying the dilution ratio enhances resolution in this ELISA system. These findings helped establish our study design. Furthermore, HRP-2Ab displayed a better linear response and calibration line for comparing Au-2Ab to HRP-2Ab (Figure 2). Even though HRP was selected as a secondary antibody in this study, the gold-nanoparticle-secondary-antibody with an LSPR signal was an acceptable preliminary experimental tool to establish an analysis plan. This technique can see through details and is more sensitive than other methods. All error bars in the following figures are determined under SD.

### 3.2 HRP Selected as the Secondary Antibody Candidate Instead of Au-2Ab for this Immunosensor

A bottom-up, layer-by-layer method was used to create our rapid-test kit.

### 3.3 Coated Bottom Layer Concentration Tested with Two Methods, and the Superadsorption Machine Method Exhibiting a Smaller Error Bar, as Determined through Standard Deviation

**Figure 3:**
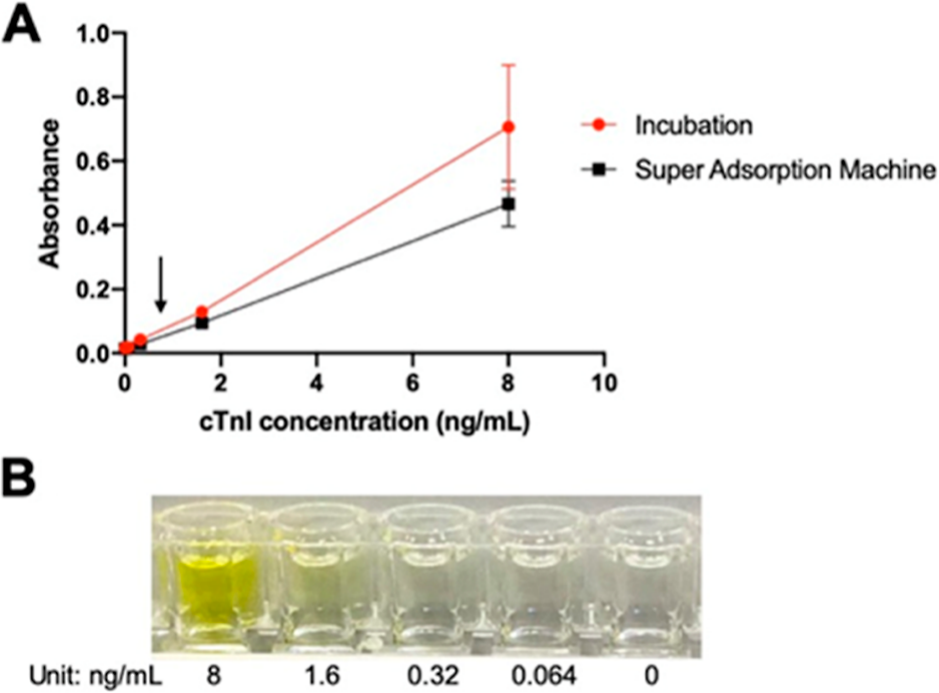
cTnI detection model. (A) Standard curve calibrated at 450 nm absorbance. The arrow indicates the area used to increase resolution and also indicates the area of human cTnI with MI, which can vary and is highly dependent on the individual; (B) color change can be seen by the naked eye; color gradually changes in response to the cTnI concentration increased from 0.064 to 8.00 ng/mL.

The coating method was studied in two ways to increase resolution. The bottom layer, coated with the primary cTnI antibody, was incubated for 1 h at room temperature or by the superabsorption machine, similar to the microwave method, for 15 min (Figure 3A).

Although it is more sensitive to dynamic range than the superabsorption machine, room-temperature incubation presented a high error bar. Thus, the superadsorption machine method was used for the bottom layer coat. The arrow in Figure 3A indicates the area used to increase the resolution for easier visualization by the human eye. Next, a picture of the well row in front of a white background was taken to focus on “tested cTnI traces that can be seen” (Figure 3B). This row was later used to design a white paper test strip in future work. As a result, the human eye could detect concentrations at 0.32–8.00 ng/mL. The yellow color was distinguishable until reaching a maximum 200 ng/mL concentration (not shown). Therefore, the lowest limit of detection (LOD) for the naked eye was 0.32 ng/mL.

### 3.4 Top Layer Tested under Seven HRP-2Ab Concentrations, Confirming the 1562.5× Dilution as the Most Efficient

**Figure 4:**
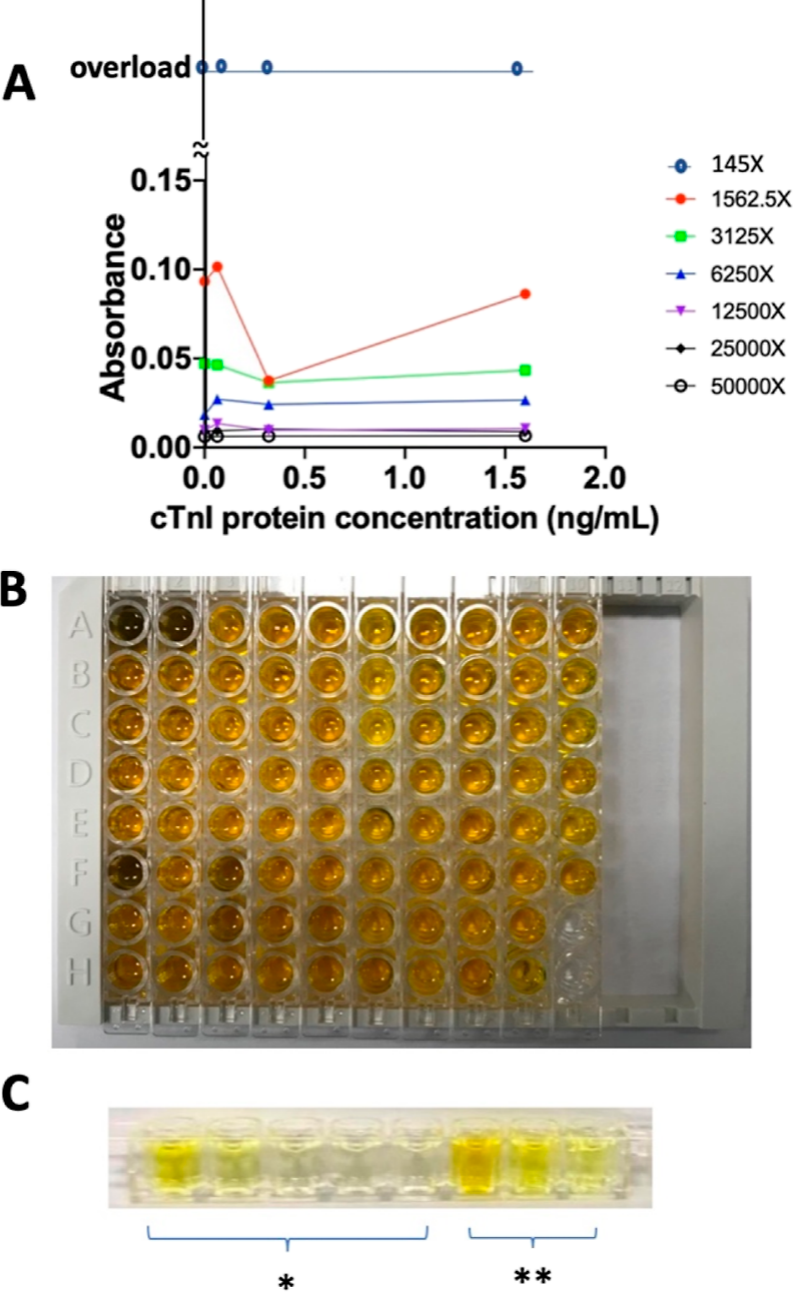
Secondary antibody dilution ratio with (A) different ratios, (B) 145× dilution, and (C) 1562.5× dilution. * Denotes that the reference wells contained 0 to 8 ng/mL standard cTnI protein; ** denotes that the sample contained human serum spiked with standard cTnI protein at three concentrations.

The top layer’s best concentration, with the secondary-cTnI-antibody-HRP as a chromophore converting agent, was studied under different dilution ratios. The cTnI protein concentrations were 0, 0.064, 0.32, and 1.6 ng/mL. The secondary antibody from the stock solution was diluted by 145×, 1562.5×, 3125×, 6250×, 12,500×, 25,000×, and 50,000×. The 1562.5× dilution resulted in a 0.32 ng/mL LOD, indicating a good responding dynamic (Figure 4). The condensed 145× solution displayed an indistinguishable dark yellow color, suggesting an overload (Figure 4B). In addition, 145× had no resolution under 450 nm (Figure 4A) and exhibited a 0.32 ng/mL LOD. These results verify that our device provides sensitivity and naked-eye detection properties at a 0.32 ng/mL LOD, the minimum cTnI concentration this device can detect under UV or the human eye. This study used 1562.5× as the secondary antibody dilution ratio in the following experiments.

#### 3.4.1 Device Preparation and Arrangement

**Figure 5:**
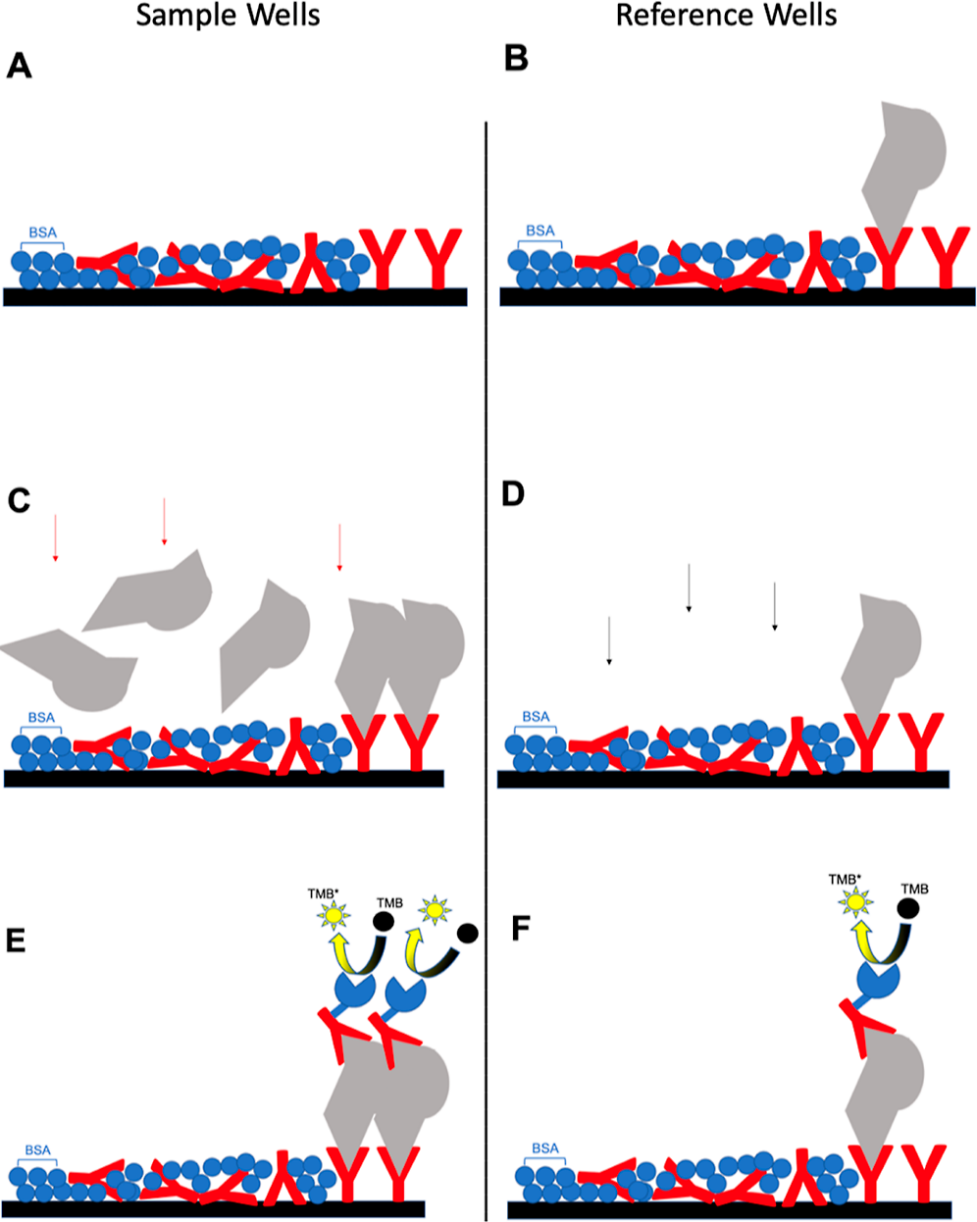
Schematic illustrating the naked-eye cTnI detection device in (A) sample wells; (B) reference wells; (C) blood loading on the sample well, with the red arrow representing blood flow; (D) PBS loading on the reference well, with the black arrow denoting PBS flow; (E,F) secondary antibody loading and chromophore acting. Different items indicate antibody (red Y-shape), BSA (blue ball), cTnI protein (gray), HRP (blue pistachio), and TMB chromophore (black ball). (A,C,E) represent sample wells, while (B,D,F) represent reference wells. The difference between sample and reference wells is that reference wells in the first step were already coated with standard cTnI protein, serving as calibration wells.

Overall, our device is bottom-up, layer-by-layer, designed and used in the following order: (1) primary antibody mixed with BSA for background blocking, (2) 0 to 8 ng/mL of standard cTnI protein is coated in the reference wells (A–E) but not the sample wells (F–H), which were then supplemented with the patient’s blood, (3) the HRP-labeled secondary antibody solution was loaded, and the chromophore TMB in the solution turned yellow, as shown in Figure 5.

There were five reference wells (A–E) next to three sample wells (F–H); all eight wells were in one row (Figure 4C). This row served as our device model, an original model that can be used as the device’s foundation in the future.

#### 3.4.2 Device Application

**Figure 6:**
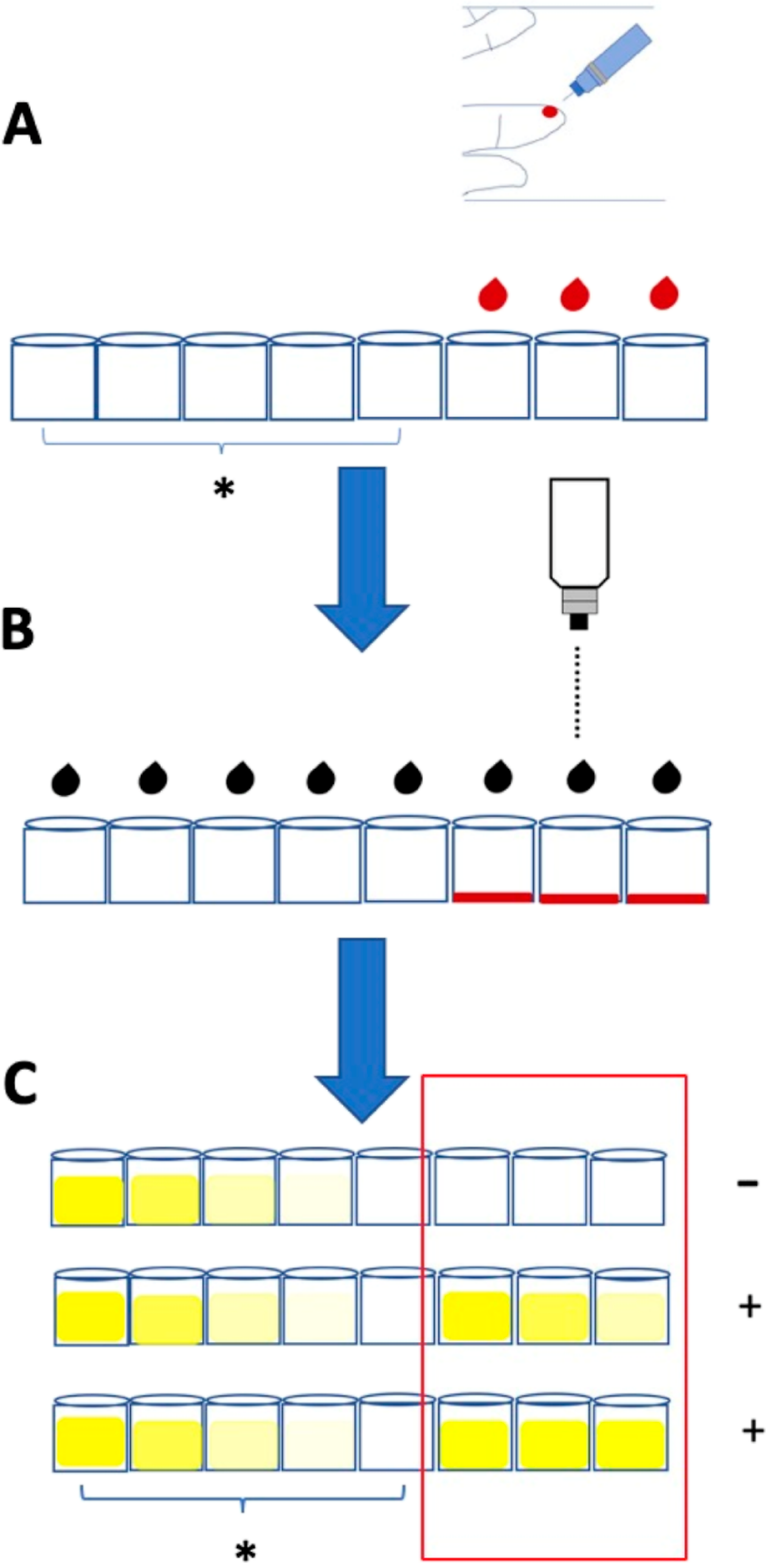
Schematic illustrating the pricking of blood at the clinic or home for MI self-diagnosis. (A) Pricking a finger and adding one drop of blood into one sample well every 30 min; * indicates the reference wells. (B) Adding the reagents. (C) Self-diagnosing MI based on the yellow pattern, where the yellow color denotes a positive diagnosis. After comparing samples to the reference well and multiplying the converting factor, the doctor or patient can determine the cTnI protein concentration at each time point. Conversion factor 15 ± 7.14 for human serum and 1.84 ± 0.76 for 10× dilute human serum. For pricking point-of-care, 1.84 ± 0.76 can be an approximate conversion factor.

**Figure 7:**
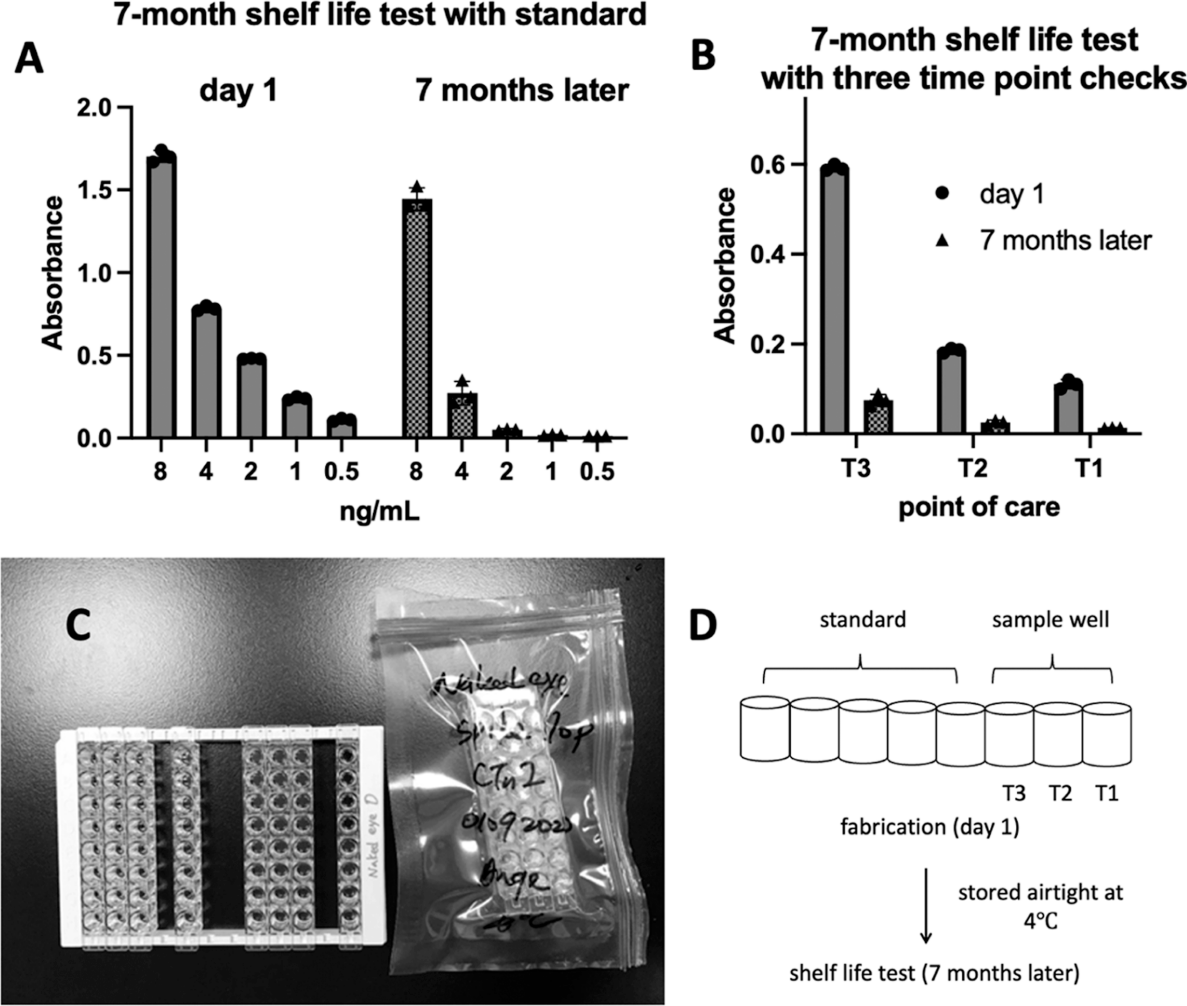
Naked-eye cTnI detection device was tested and passed 7 months of life-shelf absorbance detectable data with (A) standard and (B) sample at day 1 and 7 months later. In addition, (C) device comprises three rows in a portable, disposable bag and can be assembled onto a 96-well frame. (D) Strips are sealed in an airtight package and stored at 4 °C after the fabrication. 7-month data shows color decreasing both at the sample and standard, but with the standard curve fitting, showing that the result is repetitive and readable.

This device uses a yellow color “pattern” to denote MI diagnosis. Comparing the sample wells’ yellow patterns to reference wells indicates a cTnI protein increase in the patient. Since MI diagnosis is “cTnI protein in the circulation system that increases over time,” three sample wells should be loaded with the tested blood at three time points with the same interval. For example, blood tests can be conducted at 0.5, 1, and 1.5 h after dinner. These time point-based tests are placed into the three sample wells separately. After washing with PBS, the HRP-labeled secondary antibody solution is loaded into each. The remaining nonbinding secondary antibody is washed away, and the TMB solution is added to visualize the color of the sample and reference wells. The same concept and procedure apply to the device paper. In Figure 6, the three-time point wells denote an increase in yellow intensity, signifying a positive MI diagnosis. This device can provide self-diagnosis at the patient’s home. Furthermore, if the three time points indicate no color changes, the MI diagnosis is negative, signifying a healthy individual. This well row can then be packed airtight and preserved in the fridge for a few months. The package must be stored at 4 °C and used upon opening (Figure 7).

### 3.5 Device Tested with Human Serum for Matrix Effects, and the Results Verifying that the Device Works on Spiked, Nonspiked, and Diluted cTnI Human Serum

**Figure 8:**
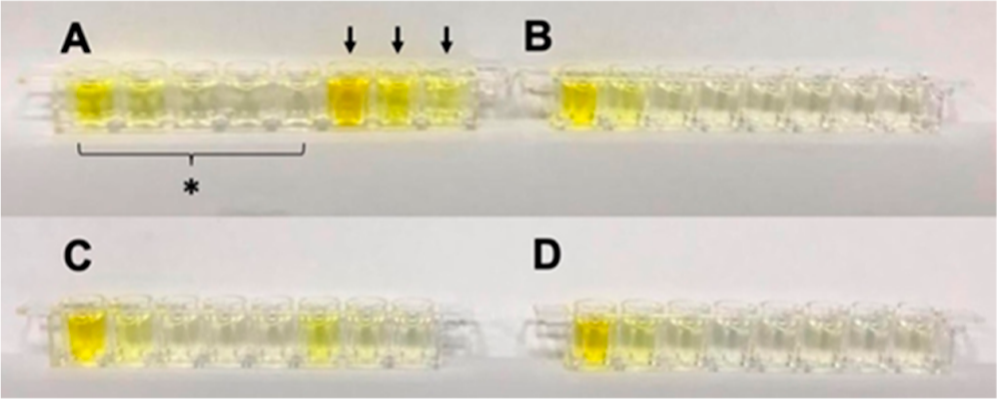
Naked-eye cTnI detection device under a human serum matrix effect (A) spiked or (B) nonspiked with cTnI protein. The 10× diluted human serum (C) spiked or (D) nonspiked with cTnI protein. A spiked sample represents a patient sample (black arrow), while a nonspiked sample denotes a healthy sample. Black arrows represent 0, 0.5, and 1 h time points (from right to left) after the patient’s first blood-pricking. Each time point blood-prick once. * indicates 0, 0.064, 0.32, 1.60, and 8.00 ng/mL of cTnI protein as the reference wells from right to left and are the same in these four well strips. This device can distinguish between an MI and a healthy patient.

This device was tested with human serum (Figure 8A) and at ten times PBS dilutions (v/v) to assess the matrix effect (Figure 8C). The sample without dilution exhibited substantial intensity and color changes compared to the healthy human sample (Figure 8A,B). Specifically, patient samples spiked with 0.08, 0.64, and 1.28 ng/mL concentrations of cTnI exhibited a significant yellow pattern shift. As shown in Figure 8B, the healthy human sample that was not spiked with cTnI exhibited no color change. However, diagnosis must still be achievable when only a limited amount of the patient’s blood is available. Therefore, the device was tested on patient samples diluted by ten times with PBS (v/v) to determine its efficacy in these conditions (Figure 8C). 10× dilute human serum shows: although the color change was not as dark as in Figure 8A, the yellow color shift was still distinguishable, allowing for MI diagnosis. Thus, our device can be clinically applied to a human serum matrix even with a shortage or dilution of human serum. This is applicable to the finger-pricking blood test design.

In the present paper, our test is shown to work in serum. The reasons I chose serum are (1) it is convenient to collect; (2) serum is commonly used in diagnostic tests; (3) this research targets specific proteins; and (4) a whole blood test (our future goal in clinic trail) cannot currently be tested in our lab, as it requires specific regulations.

Since our test works in serum, it should also work in plasma, which only contains extra proteins. ELISA is a protein-specific technique; even with extra proteins, ELISA can still work for cTnI binding. Similarly, we would expect that the test should work on whole blood with buffer dilution. In future work, we would like to confirm if the whole blood works in the clinical trial.

**Figure 9:**
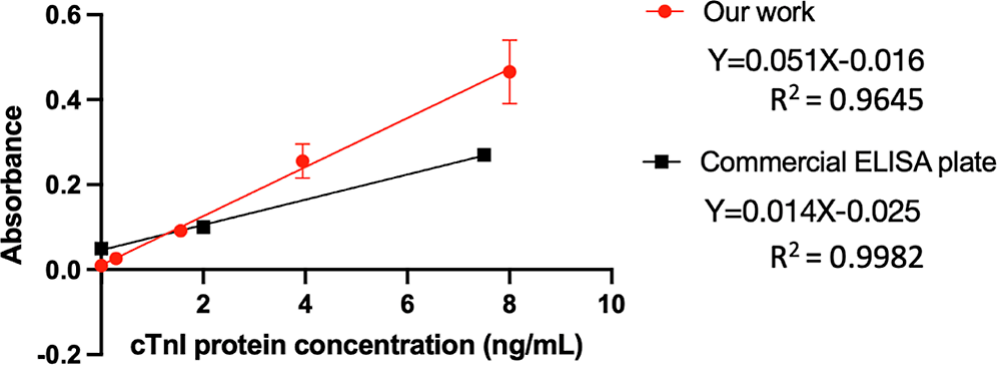
Standard curve of cTnI protein between the proposed test strip and a commercial ELISA plate. The *R*^2^ of the proposed test strip is 0.9645. The linearity is (*R*^2^)^1/2^ = 0.9821.

Our device exhibited significant advantages compared to the commercial cTnI ELISA plate (Table 1). The proposed device was compared to the commercial cTnI ELISA plate manufactured by BioCheck, Inc. The standard curves are shown in Figure 9, while other factors are shown in Table 1. The proposed device exhibited a better LOD, a shorter detection time, and easier use in clinic or home settings. The proposed device has the following advantages: (1) no detector is required, (2) portability, and (3) a small reagent volume. Moreover, compared to the commercial plate that requires a UV–vis detector, the proposed device is easier to use and enables self-diagnosis.

**Table 1:** Comparison between the Proposed Device and the Traditional cTnI ELISA Plate

	proposed device	traditional cTnI ELISA plate
accessibility	can be used at home	can only be used by a trained scientist
naked-eye detection	more convenient; reader is not required	reader is required
sensitivity^a^	high (slope = 0.051)	low (slope = 0.014)
trace detection	improved LOD: 0.31 ng/mL	LOD: 1.79 ng/mL
detection range	larger 0.31–200 ng/mL	1.79–75 ng/mL
detection time	faster 70 min	120 min
storage temperature	4°C	4°C

a Sensitivity is distinguished by the slope in Figure 9.

## 4 Conclusions and Future Work

Our study developed a device that can be used for self-diagnosis at home or pharmacokinetic clinical studies. Statistically, many patients die of MI in their homes, at work, or when driving home. Due to the relative business of their daily lives, these patients cannot afford to spend additional time at the clinic. Similar to patients measuring their blood sugar daily, this device allows patients to check their cTnI level. This device can be modified to be portable, allow daily tests, and track cTnI levels to determine heart health, significantly helping patients with heart disease.

Identifying MI symptoms can be challenging, as patients may overlook these signs as just feeling tired, needing a break, or having only slight chest pain. Patients do not consider these easily overlooked symptoms severe enough to warrant a hospital or clinic visit. As evidenced by blood sugar disease, although these symptoms are not urgent, they gradually damage the patient’s health. Thus, a self-diagnosis device is essential. However, various questions remain: “How much cardiac troponin is typically in the blood following MI?” “How quickly does this occur?” These variables depend on the individual. Some patients experience a MI within a week.

When a patient’s cTnI level gradually increases, it is often read as an injured cardiac muscle releasing cTnI into the blood (a clog may be imminent). So, our device uses a pattern to define a patient’s risk for MI. This pattern can also be used to diagnose heart injuries or diseases related to the cTnI protein, such as systemic amyloidosis and cardiovascular disease. Moreover, this diagnosis method can be used for all cardiovascular diseases that release cTnI protein into the blood (Figure 13). Our proposed devise provides reliable self-diagnosis and only requires blood at three time points; then, the patient adds PBS and the reagent in series to complete the test, similar to a COVID-19 test.

Concerning the pharmacokinetic study, with the converting factor, this device also supplies a precise cTnI concentration at a specific time, which is beneficial for determining how much medicine to administer. For example, heparin is an antithrombosis medicine. When the cardiac muscle is injured, blood clogs on-site. Heparin can release the clog and facilitate the unclogging process. With this device, a patient injected with heparin can be simultaneously measured for cTnI protein, and the clinician can determine if an additional dose is needed (Figure 11). This information can be recorded and gathered during drug combination studies, not just for one medicine at a time (Figure 12). In combination drug studies, this device can customize the best medicine combination for a patient since every patient exhibits different medical histories, such as a stent, bypass, and balloon.

### 4.1 Clinical Usage in Pharmacokinetic and Pharmacodynamic Studies

**Figure 10:**
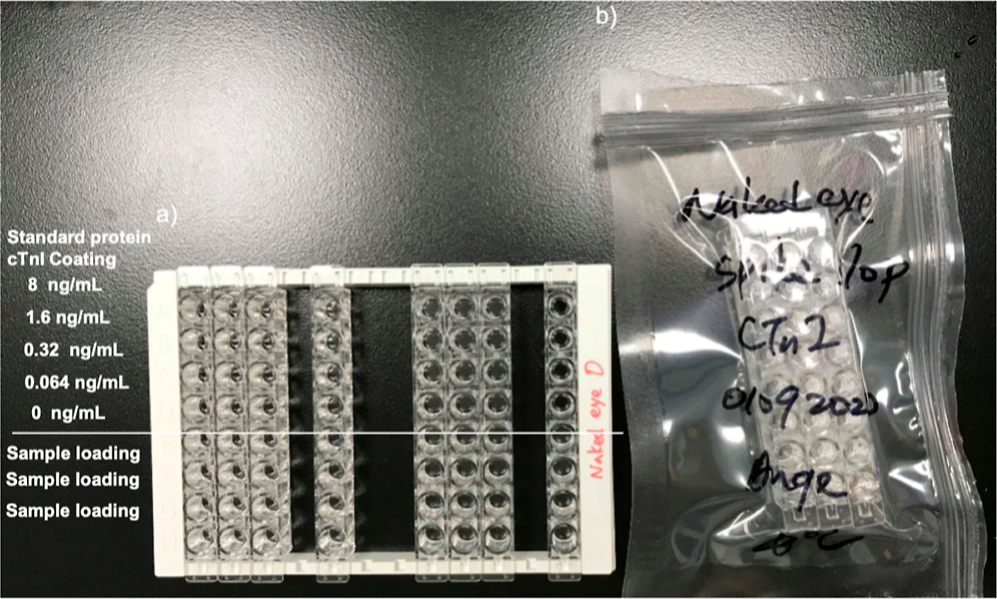
For pharmaceutical study. (A) Portable and can be assembled in a 96-well frame for high-throughput screening; (B) sealed package. Methodology advantages: (1) easy fabrication, can easily apply mass production; (2) portable, easy to carry and use.

**Figure 11:**
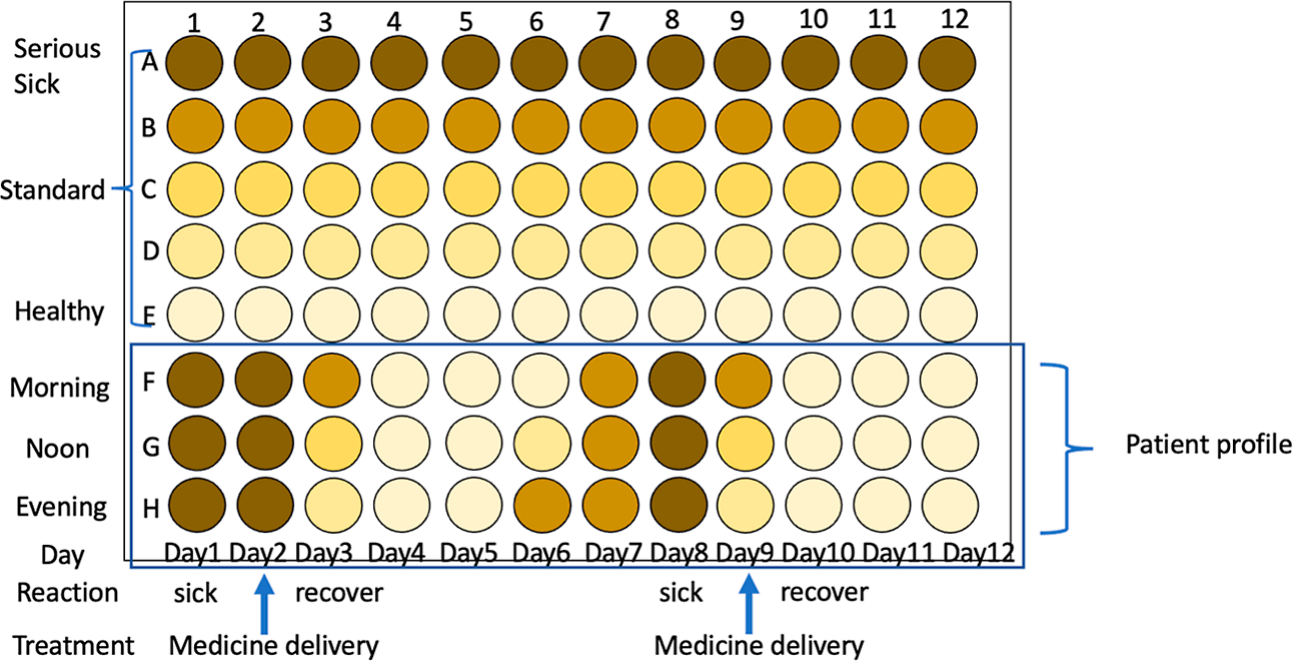
Tracking pharmaceutical kinetics and patient profiles. Advantages: (1) easy to track patient profiles; (2) easy to check medicinal pharmacokinetics; (3) portable, easy application; (4) only requires a small sample loading amount; (5) delivers medicine accurately; and (6) medicine economics.

**Figure 12:**
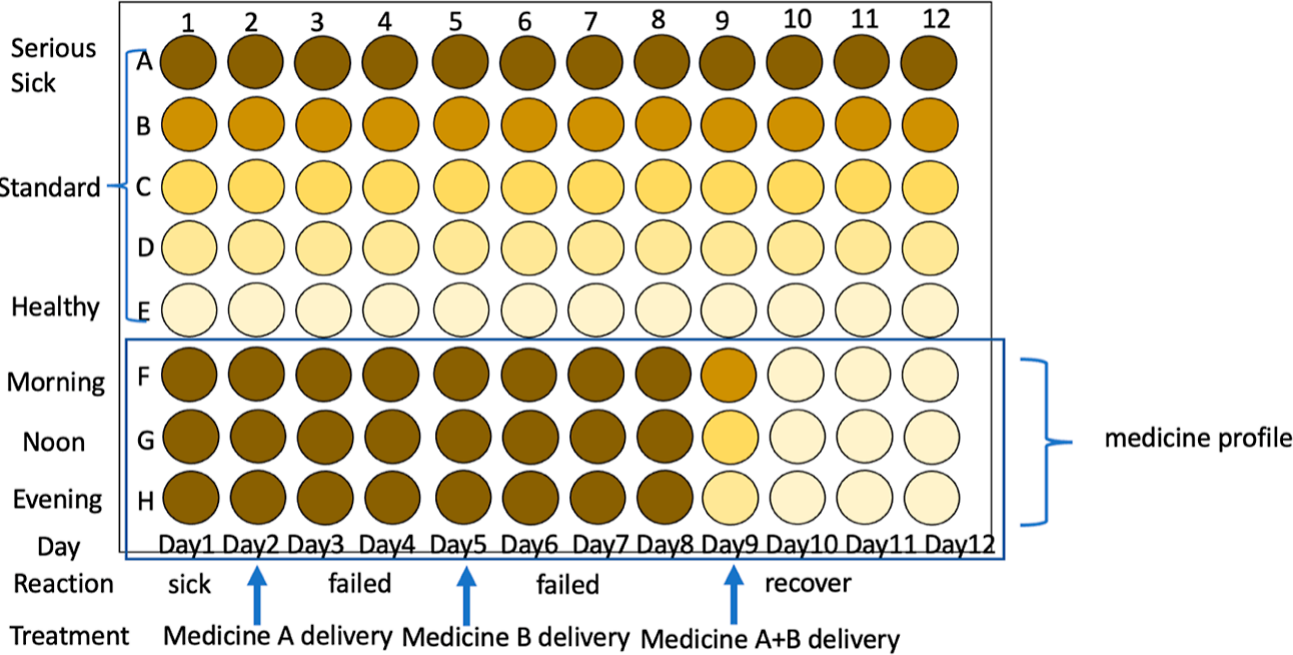
Establish medicine profiles for selection based on pharmacodynamics. Advantages: easy to establish medicine profiles with different doses, medicine selections, examiner ages, and combinational drugs.

During pharmacokinetic and pharmacodynamic studies, sealed devices can be used in the following applications (Figure 10) and for tracking pharmaceutical kineticss and patient profiles (Figure 11). The proposed device can establish a medicine profile for selecting the right medicine based on pharmacodynamics for precise targeting (Figure 12).

**Figure 13:**
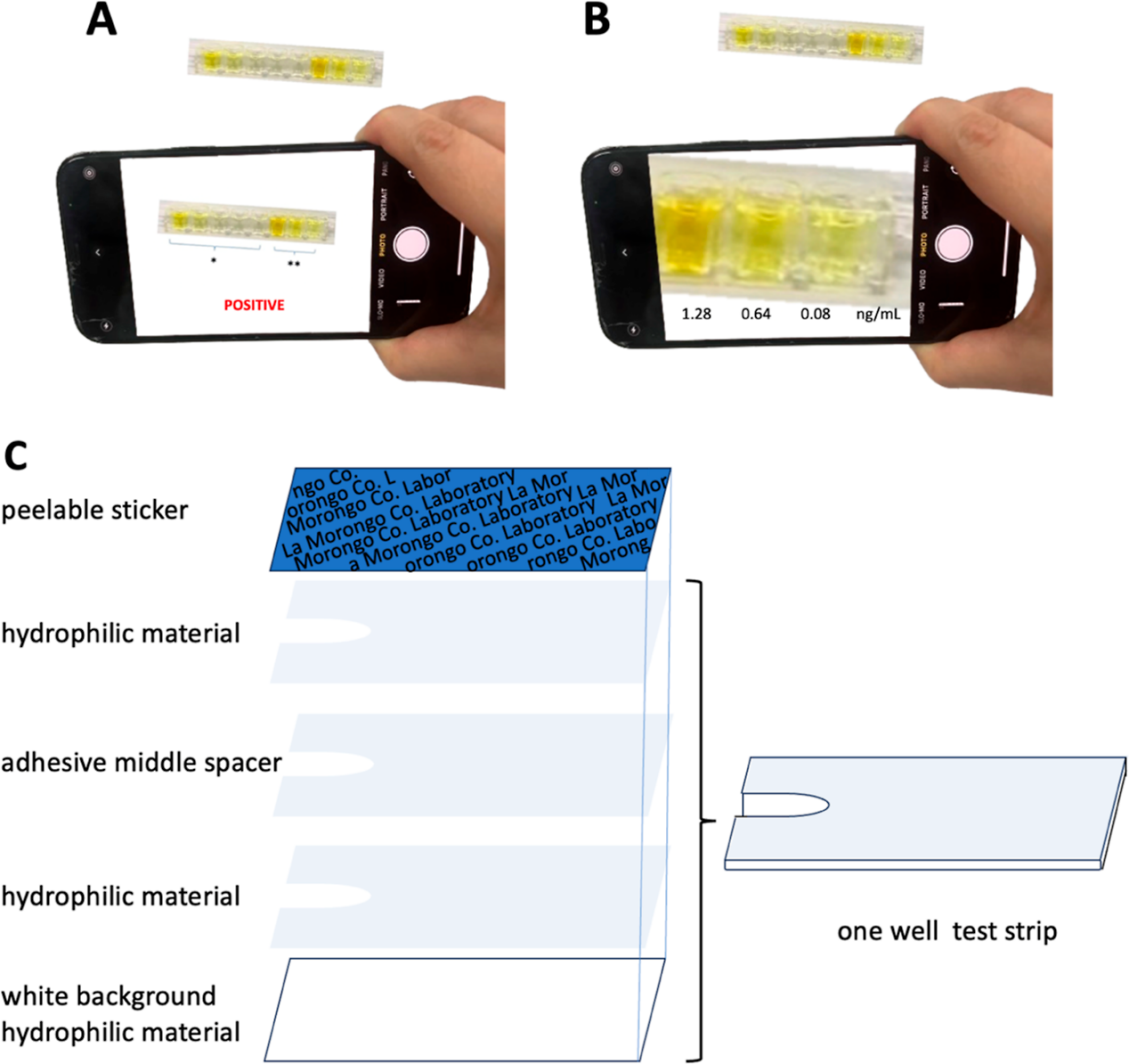
Future work schematic diagram by using an app to (A) rapid-test positive/negative diagnosis and (B) reading the concentration by image pixel between the sample and the standard; (C) fabrication of one well test strip. By loading blood into white background hydrophilic material layer test strip, we can both fix the blood volume and decrease the surface tension to improve the image quality. Extending one well test strip to an 8 well array, patients can take pictures with a cell phone, diagnose, gather preclinic data, track MI progression, record medicine doses, analyze medicine digestion, and design customized combinational drugs with an artificial intelligence (AI) app.

In future work, we plan to invent an app for positive/negative tests (Figure 13A), and with the comparison of the pixels in the enlarged color image, the app could tell the concentration of diagnosis (Figure 13B). Patients could take and upload daily pictures, and the app would allow for analyzing, recording, and tracking results to improve public health. Similar to blood sugar test kits, with the design of the layer test strip (Figure 13C), the blood loading volume can be fixed. This app could further use AI to build the loop between clinical opinion and patient feedback, collecting big data directly from the public point of care. The vials we used to test are an 8-vial array in a line, of which the first five are standard and the last three are for the sample. By fitting the color of the sample to the color of the standard, it gets the result. Since the 8 vials are close to each other, it is unlikely the light can interfere with the reading because every vial has the same background noise. The background light is the common background noise. That is, to protect against very rare instances, we may try to design the app using image processing techniques so that the app can detect when lighting properties differ significantly across the vial array. In such cases, the app would try to correct for the different lighting.
